# Genome-Wide Assessment of Efficiency and Specificity in CRISPR/Cas9 Mediated Multiple Site Targeting in Arabidopsis

**DOI:** 10.1371/journal.pone.0162169

**Published:** 2016-09-13

**Authors:** Brenda A. Peterson, David C. Haak, Marc T. Nishimura, Paulo J. P. L. Teixeira, Sean R. James, Jeffery L. Dangl, Zachary L. Nimchuk

**Affiliations:** 1 Department of Biology, University of North Carolina at Chapel Hill, Chapel Hill, NC, United States of America; 2 Curriculum in Genetics and Molecular Biology, Carolina Center for Genome Sciences, University of North Carolina at Chapel Hill, Chapel Hill, NC, United States of America; 3 Department of Microbiology and Immunology, University of North Carolina at Chapel Hill, Chapel Hill, NC, United States of America; 4 Howard Hughes Medical Institute, University of North Carolina at Chapel Hill, Chapel Hill, NC, United States of America; 5 Department of Biological Sciences, Virginia Tech, Blacksburg, VA, United States of America; 6 Department of Plant Pathology, Physiology, and Weed Science, Virginia Tech, Blacksburg, VA, United States of America; East Carolina University, UNITED STATES

## Abstract

Simultaneous multiplex mutation of large gene families using Cas9 has the potential to revolutionize agriculture and plant sciences. The targeting of multiple genomic sites at once raises concerns about the efficiency and specificity in targeting. The model *Arabidopsis thaliana* is widely used in basic plant research. Previous work has suggested that the Cas9 off-target rate in Arabidopsis is undetectable. Here we use deep sequencing on pooled plants simultaneously targeting 14 distinct genomic loci to demonstrate that multiplex targeting in Arabidopsis is highly specific to on-target sites with no detectable off-target events. In addition, chromosomal translocations are extremely rare. The high specificity of Cas9 in Arabidopsis makes this a reliable method for clean mutant generation with no need to enhance specificity or adopt alternate Cas9 variants.

## Introduction

Many gene families that regulate key processes are highly redundant and spread across diverse chromosomal locations in plants. To understand gene function this necessitates the ability to simultaneously target and mutate distinct loci in a highly specific manner without affecting other genes. The CRISPR/Cas9 system has emerged as a powerful tool to create targeted mutations in plants [1]. By co-expressing multiple guide RNAs (gRNAs) with the Cas9 enzyme in plants efficient multiplex mutation of gene families can be achieved. Off-targets can be predicted computationally based on divergence from the desired on-target site [2]. Current data suggests that off-target mutagenesis rates in plants are low [3, 4] or undetectable, however this assessment is based on data from small scale targeting events. In many non-plant systems the use of Cas9 in targeted mutagenesis is off-set by considerable rates of off-target events. Several approaches targeting either Cas9 or the gRNA have been created by different groups that lead to reduced off-target rates in non-plant systems [5–7]. It is unclear if these approaches would be necessary in plant based systems. In higher order multiplex experiments the potential for off-target effects and chromosomal translocations increases significantly and has implications for the interpretation and confirmation of phenotypes. To explore these issues we performed whole genome re-sequencing of Arabidopsis plants targeting 14 genomic loci at once and assessed on-target and off-target Cas9 efficiencies and translocation events.

## Results

Our lab is interested in the function of redundant peptide encoding genes that contribute to development. To facilitate the higher order multiplex mutation of plant peptide encoding genes (see methods for details), we assembled gRNA expression units into “stackable arrays” targeting up to 14 sites at once. We used our stackable array approach to target peptide families from the 11 member *GOLVEN* (*GLV*/*RGF*/*CLEL*) gene family which encode peptides that regulate several aspects of root stem cell development, but have not been studied in above ground tissues [8–10]. We targeted the six *GLV* genes that are expressed in aerial tissues, and *CLE18*, (S1 Table), which gives rise to a GLV-like peptide in aerial tissue [9, 11]. For each of the seven genes we selected two gRNA targets sites per gene using standard Cas9 targeting requirements. gRNA units, each consisting of a gRNA and Arabidopsis *U6* promoter, were synthesized in clusters of four (see methods), and then stacked together to generate a vector harboring a total of fourteen gRNA units. This stacked array was cloned into the custom *pCUT3* binary vector and transformed into wild-type Col-0 plants. We obtained multiple T_1_ plants, and first screened plants for editing of *GLV1* by analyzing Sanger sequenced PCR fragments from T_1_ leaves to confirm Cas9 function in these lines (S2 Table). Although we typically observe high numbers of plants with editing activity using our system, we have also noted a bias in many cases against recovery of T_1_ plants containing editing activity when targeting genes that regulate embryogenesis or essential functions (Nimchuk, unpublished). In this case we observed evidence of *GLV1* editing in only one plant from the six we surveyed, considerably lower than the near 100% efficiency we typically observe when targeting single non-essential genes. Details on the phenotypes arising from the stable septuple mutant plants will be presented elsewhere.

Leaves are composed of a large number of multiple cells types. In somatic *Cas9* expressing plants each leaf cell is capable of having a distinct editing event. Thus a single leaf contains an array of editing events. Therefore, to assess genome wide editing efficiency we sampled whole leaf tissue pooled from 48 T2 plants to maximize the spectrum of detectable mutation types. We then purified DNA from the pooled leaves and subjected them to resequencing on the Illumnia HiSeq 2000 platform. Reads were mapped to the TAIR10 reference genome using BWA v 5.7 [12] and variants were called using FreeBayes v0.9.10 [13] and confirmed visually using the Integrated Genome Viewer v2.3.60 [14] (S1 Fig). Sequencing reads were generated across two lanes of Illumina. Editing events were only called if they occurred in both lanes and average coverage for on-target and off-target sites was 100X per lane (split pane in S1 Fig indicates each lane).

**Fig 1:**
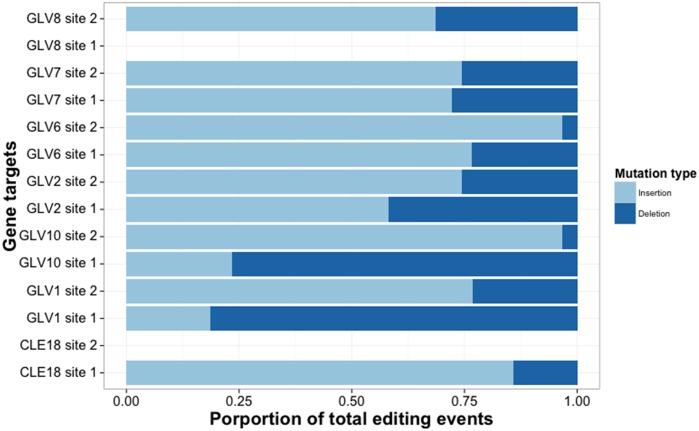
Insertion bias in editing events. Bias in insertions relative to deletions within editing events (likelihood ratio test, X^2^ df = 1, p <<0.001). Light blue bars indicate insertion events, dark bars indicate deletion events. No editing activity was detected for either CLE18_2 or GLV8_1.

Cas9 preferentially cuts at the -3 position relative to the PAM sequence in a target site resulting in insertion/deletion events [15]. We therefore considered indel events at or near this location, rather than base substitutions, as being clear evidence of Cas9 activity rather than sequencing errors. We note that large deletion events created by dsDNA breaks at the -3 position followed by DNA deletion prior to repair could render individual reads un-mappable and may be selected against in our assembly. However these are expected to be infrequent as previous work has shown that Cas9 induced events are strongly biased to smaller indel events, with larger indels being increasingly rare [3]. As such if anything, our method will underrepresent on-target editing events. We first assessed the frequency of on-target editing efficiency in our multiplex system. Out of the 14 gRNAs transformed, two did not show any evidence of cutting (Table 1). This is comparable with frequencies of single gRNA expression in our lab (Nimchuk lab, unpublished). Of the remaining 12 gRNAs, on-target efficiencies ranged from 33% of sequence reads containing Cas9-consistent editing, to 92%. There was no clear correlation between editing efficiency and strand bias in the dataset. Both gRNAs that failed to edit targeted the minus strand, however several gRNAs that targeted the minus strand promoted editing at a rate equal to or greater than some of the gRNAs that targeted plus strands. We observed a significant bias in insertions over deletions (Fig 1, S3 and S4 Tables) which was not explained by strand, site, or distance from PAM (S4 Table), however, two genes (*GLV1* and *GLV10*) showed the opposite effect.

**Table 1:** On-Target Rates.

Site	Gene	Target Sequence	Total Events^a^	Total Reads	Percent^b^
CLE18_1	AT1G66145.1	tcgaggaaaacaatatcgac	113	217	52.1
CLE18_2	AT1G66145.1	aaaacaatatcgaccggtct	0	0	0
GLV1_1	AT4G16515.1	cttctttggacgtcttcaa	124	181	68.5
GLV1_2	AT4G16515.1	gagaaaggaggcgggcgtt	121	183	66.1
GLV2_1	AT5G64770.1	tgcgacgaggaaagacttg	86	231	37.2
GLV2_2	AT5G64770.1	aatagaacgctattggttg	121	219	55.3
GLV6_1	AT2G03830.1	gctagtgcacaaagaaaga	219	251	87.3
GLV6_2	AT2G03830.1	tggagttacaagcaataaaa	90	267	33.7
GLV7_1	AT2G04025.1	tgatgaaaaagatgatacg	90	271	33.2
GLV7_2	AT2G04025.1	ctgaaacgaagatgaagag	149	198	75.3
GLV8_1	AT3G02242.1	atcaatcccaaaaagaagaa	0	0	0
GLV8_2	AT3G02242.1	aagacaagcaataaagctg	185	243	76.1
GLV10_1	AT5G51451.1	aaggatcattgaagcaaca	167	250	66.8
GLV10_2	AT5G51451.1	gataatctgcaaataagag	239	259	92.3
**Total**			1704	2770	61.5

^a)^ Total number of editing events, including both insertions and deletions.

^b)^ Number of editing events per number of reads analyzed.

We next computed the most probable off-target sites for each gRNA using the CRISPR-P webtool interface (http://cbi.hzau.edu.cn/crispr/ [16]). This site uses algorithms based on the Hsu *et al* assessment of Cas9 specificity and gRNA-target mismatch tolerances in over 700 gRNAs tested in human cell lines [17], and allows for sites encoding a non-canonical NAG PAM site as well as the prototypical NGG PAM site for Cas9. We limited our analysis to the top 20 scoring off-target candidates for each on-target site but see below for other sites). For some on-target sites there were less than 20 off-target sites that matched the required criteria. A total of 178 off-target sites were assessed which were collectively covered by nearly 43500 reads in our dataset with an average read coverage of 100X (S5 Table). Surprisingly we found no evidence of Cas9-induced indel events at any site in the dataset. To screen for indel events across the genome at sites not predicted to be potential off-target site we first filtered our Freebayes derived indel set by quality and depth (to remove highly repetitive regions). From this data set we recovered 4 additional sites (not including the intentional Cas9 mutations) that had a minor allele frequency greater than 3%. Sequencing of three of these sites in different non-transgenic parental Col-0 plants lines revealed similar indels to our Cas9 containing line in some of our Col-0 plants for two of the sites. For the third we found a slightly larger deletion among the six sequenced plants that were at the same location (S7 Table) suggesting that this location is a hotspot for deletion events in Arabidopsis. We were unable to get quality sequencing data back for the 4^th^ site among our non-transgenic plants despite several attempts. These data indicate that the deletions events we noted in the non-predicted off-target sites likely arose by natural divergence from the reference Col-0 in our local stocks prior to the introduction of Cas9.

**Fig 2:**
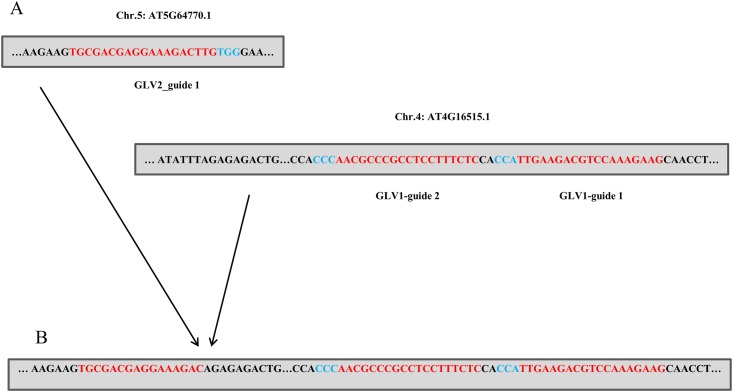
Translocation Events Translocation between *GLV2* and *GLV1*. Target sites prior to (A) and after (B) CRISPR induced translocation. Red sequences indicate target sites while blue sequences indicate PAM sites.

We next queried our data for evidence of translocation events using IGV v2.3.60. In multiplex gene targeting the prevalence of multiple dsDNA breaks in the same cell has the potential to generate translocation events. In mammalian systems this property has been exploited to create cell lines that mimic cancer causing chromosomal aberrations and has been noted in several studies [7, 18, 19]. To avoid sequencing errors we considered reads that originated in one on-target site and were contiguous with another on-target site and were supported by multiple reads on both sequencing lanes. Considering just the 7 genes targeted, and not the individual gRNAs, there is the potential for 42 different translocation events to arise between different targeted genes for simple translocation scenarios. Despite this, out of all the sites monitored we found only two independent events, both of which generated translocation events that occurred between *GLV2* gRNA target site 1 and *GLV1* gRNA sites 1 or 2 (Fig 2). These events arose in 1–2% of the total reads spanning both areas. Therefore Cas9 induced translocation events arise at a very low frequency in Arabidopsis.

## Discussion

Considerable effort has been made to improve the on-target efficiency of Cas9 in genome editing systems in non-plant systems. Our data demonstrate that the original Cas9 system based on the wild type *Streptococcus pyrogenes* Cas9 is already specific enough to make off-targeting events undetectable in Arabidopsis. Our work supports previous data using smaller scale targeting and suggests that there is no inherent need to adopt different Cas9 variants in Arabidopsis genome targeting systems. While it is formally possible that there may be some sites for which off-targets may be efficiently cleaved for some gRNAs, our data suggests that this will be very rare. Consistent with this off-target rates at computationally predicted sites were undetectable and the other indels surveyed in our system likely arose in our stock line of Columbia-0 due to spontaneous mutations rather than Cas9 editing activity [20]. In theory, if the average off-target cleavage rate of Cas9 in Arabidopsis were to be a mere 0.01% among our computationally predicted off-target sites we would still expect to find 4–5 such events in our total off-target dataset, and at 0.1% we would expect 43 events. The lack of detection of these events strongly suggests that Cas9 off-target effects are negligible in Arabidopsis. This contrasts with transcription activator-like effector nuclease (TALEN) and designer transcription activator-like effector (dTALE) systems which have been shown to result in off-target editing in plants at low rates.[21–23] To our knowledge no off-target editing has been reported using zinc finger nucleases (ZFN) [24]. Despite this, the the CRISPR/Cas9 system still offers advantages in ease of design compared to ZFNs. In addition, we find that Cas9-induced translocation events are extremely rare in Arabidopsis. This observation suggests that higher order multiplex genome editing will not likely be hindered by chromosomal aberrations. Clark and Krysan showed in a small scale survey of T-DNA mutants that chromosomal translocation events could be detected in 11/64 T-DNA insertion lines [25]. Our data suggest that translocation events induced by Cas9 are lower than the frequency of translocations that arise during T-DNA transformation in Arabidopsis [25]. In addition combining multiple T-DNA insertion events with common sequence similarity in the T-DNAs can stimulate recombination upon crossing [26]. As such higher order mutant creation with Cas9 might be a cleaner genetic system than T-DNA based crossing. It is likely that some translocation events are deleterious and are selected against and might not be identified in our analysis. However some translocation events are tolerated in Arabidopsis and we view it likely that dsDNA breaks induced by Cas9 are efficiently coupled to repair to select against translocation events. On-target mutant screening strategies based on PCR amplification using primer sets flanking gRNA on-target sites will further counter-select against rare chromosomal events.

While our findings indicate that Cas9 is highly specific in Arabidopsis it is not clear how these results would relate to other plant species. Detailed studies on the specificity of Cas9 in other plant species will require further research. It is not clear why Cas9 is highly specific in some systems and not others. Our study of the *GLV* gene family in Arabidopsis is likely to be representative of Arabidopsis, and not an effect of the *GLV* family specifically. Our gRNAs are diverse in sequence and are computationally predicted to have potential off-target sites throughout the genomes. As such, factors such as off-target chromosome location, methylation status, and nucleotide mismatch number and position would be expected to be variable and represented our dataset. Despite this we find no off target events. We have noticed that Cas9 accumulates to very low levels in both Arabidopsis and tobacco with our system despite being codon optimized for plants and being expressed from the *UBIQUITIN10* promoter (ZN unpublished). High levels of Cas9 are correlated with the formation of energetically less favorable off-target events in others systems [27]. As such, our favored hypothesis is that low Cas9 levels in Arabidopsis limit off-target events to highly specific target pairing events. This hypothesis will require further experimentation to fully test. Our data presented here demonstrate that Cas9 is an extremely precise enzyme for genome editing in Arabidopsis and that off-targeting and chromosomal aberrations are not likely to be complications in multiplexing experiments.

## Materials and Methods

### CRISPR/Cas9 vector system construction and design

A dual maize and Arabidopsis optimized *Cas9* gene was gene synthesized with a Hemagglutinin (HA) tag linker at the C-terminus fused to the N7 localization tag, which directs nuclear localization of expressed fusion protein [28], by GeneArts. This was subcloned into a modified pENTR3C vector (Invitrogen) containing an upstream omega translational enhancer sequence and then recombined into a pMOA vector series behind the Arabidopsis *UBQ10* promoter sequence [29] to create the pCUT binary series. To create the pCUT-G gateway compatible series, Gateway cassettes (Invitrogen cat# 11828–029, rfB) were released from pBluescript with EcoRV and ligated into pCUT vectors linearized with PmeI. The resulting plasmids’ full-length sequences were confirmed via HTS.

For multiplex stackable array construction, a digital template of 4 gRNA units was created in LaserGene and saved as a.gb file with 19Ns replacing the putative target sequence in each gRNA unit. For each gene of interest to be targeted, Cas9 target sites were selected using standard bioinformatics criteria [30]. Our template contains a transcriptional start site G in each gRNA unit between the *U6* promoter sequence and the gRNA. We have found that this renders the requirement for an upstream G in the standard G-19/20N-NGG consensus site not strictly necessary and that N-19/20N-NGG sites will also be targeted efficiently (S2 Fig). To be conservative, where possible G-19/20N-NGG sites were selected. Target sites were then pasted into each gRNA unit to create a digital array that was then synthesized (GeneArts; Regensburg, Germany). Stacked arrays were cloned together by restriction enzyme digestion of one array (donor) into a pMA gRNA array linearized with EcoRV (recipient), thus destroying the PmeI sites in the donor but preserving the flanking PmeI sites in the recipient (S3 Fig). Stacked arrays were then cloned into pCUT3 using the Mighty Mix ligation system (Takara).

### Transformation of Arabidopsis

Plant expression vectors were transformed into an *Agrobacterium tumefacians* recA- strain via triparental mating (strain C58, gift from Stephen Farrand). Wild type *Arabidopsis thaliana* Col-0 plants were grown in a growth chamber with constant light at 23°C. Plants were transformed using the floral dip method [31]. T_1_ seed derived from plants transformed with binary pCUT3 were selected on B5 media lacking sucrose and containing 100mg/L kanamycin (S6 Table).

### Sanger sequencing analysis of CRISPR events

Plant and bacterial DNA were extracted as previously published [29]. Forward and reverse primers were designed to anneal to genomic DNA sequences at approximately 250 bp flanking the putative clipping sites (S2 Table). PCR was performed using PfuUltra II Hotstart 2X Master Mix (Agilent Technologies, Inc). After amplification the PCR products were purified using the Zymo Clean and Concentrator Kit (Zymoresearch, USA). For direct sequencing of PCR products, the same primer set for amplifications were used. For sequencing of cloned PCR products, PCR fragments were cloned directly into pCR2.1 using the manufacturer’s recommendations for the Zero Blunt Topo cloning kit (Life Technologies), and sequenced using M13F and M13R primers. In both cases DNA traces were compared to wild type sequences using the SeqMan function from LaserGene analysis package. For directly sequenced PCR products, deviations from a consensus trace that originated near the -3 position relative to the NGG PAM site, the known site of Cas9 cutting, were scored as evidence of genome editing in somatic tissues relative to wild type traces.

### Illumina DNA preparation, sequencing and bioinformatics

A total of 150 ng of each plasmid was used to prepare libraries following the TruSeq DNA prep protocol (Illumina). In brief, DNA was sheared to a mean size of 400 bp by ultrasonication (Covaris), end-repaired, adenylated and ligated to barcoded adapters. Adapter-ligated molecules were then submitted to 14 cycles of PCR using the KAPA HiFi DNA polymerase (KAPA Biosystems). The amplified libraries were pooled and sequenced on an Illumina HiSeq2500 instrument to generate 48 bp paired-end reads. Approximately 230,000 paired-reads were obtained for each library. The Velvet software package [32] was used to assemble the plasmids into single contigs using k-mers that ranged from 44 to 46 nt. Sequencing data has been deposited in the SRA database under project SRP073942.

### Statistical analysis

We used the glmer function in the lme4 package developed for the R computing platform to generate logistic regression models to test for bias in event types. All fitted models included total editing events as the response variable and gene as a random effect. Models with differing fixed effects (insertions, deletions, site, and distance from PAM) were used for multi-model inference (MMI). The best fit models were chosen based on the change in AICc (Akikiae information criterion corrected for small sample size) and likelihood ratio test (S4 Table).

## Supporting Information

S1 Fig On-target editing events for GLV2, image from the Integrated Genome Viewer v2.3.60 of mapped sequencing reads. Sequencing lanes were kept separate and are displayed in different panels (upper panel is lane 1, lower panel is lane 2). Blue symbols represent insertions and dashes represent deletions in the mapped reads. In this view, the editing events can be seen for both site 1 and site 2. See https://www.broadinstitute.org/igv/ for a complete description and guide for the use of the IGV.
(TIF)

S2 Fig Stackable array template. A 4 gRNA unit array. Each unit contains an Arabidopsis *U6* promoter (blue), target site pasting location (poly n streach), and gRNA backbone with poly T terminator (yellow). Each unit is separated by a unique blunt restriction sites (underlined) in the following order: SmaI, SnaBI, HpaI, NaeI, EcoRV (S3 Fig). The entire array is flanked by PmeI sites on either site (Red). Higher order arrays can be synthesized but take considerably longer than a 4 unit array. Note: the poly n stretch is proceeded in each case by a transcriptional G. Each poly N stretch is replaced with the desired genomic target site, both 19 and 20 nt target site will work.
(TIF)

S3 Fig Stackable gRNA array platform for use with the pCUT vector series. A) The *pCUT* binary vector system. A plant codon optimized HA-tagged nuclear localized (N7) Cas9 is expressed from the Arabidopsis *UBQ10* promoter. A transformer boosting sequence (*TBS* [1]) separates the *Cas9* expression cassette from a unique PmeI site (red). OCS; octopine synthase terminator. B) Strategy for array stacking. Array gRNA units (A-L), are synthesized or cloned in groups of four containing flanking PmeI sites (red) and unique blunt cutting sites (blue). Arrays are stacked as PmeI fragments cloned into unique Sma1 or EcoRV blunt sites iteratively to preserve the flanking PmeI sites. Example shows stacking of three 4 unit arrays to create a 12 unit stacked array. Reference 1: Hily JM, Singer SD, Yang Y, Liu Z. A transformation booster sequence (TBS) from Petunia hybrida functions as an enhancer-blocking insulator in Arabidopsis thaliana. Plant cell reports. 2009;28(7):1095–104. doi: 10.1007/s00299-009-0700-8. PubMed PMID: 19373469.
(TIF)

S1 Table Genomic target sites for Cas9. (DOCX)

S2 Table Primers used for PCR amplification and sequencing. (DOCX)

S3 Table On-Target Rates. (DOCX)

S4 Table Comparison of model fits for insertions, deletions and additional explanatory variables. (DOCX)

S5 Table Off-Target Rates. (DOCX)

S6 Table pCUT binary vectors. (DOCX)

S7 Table Indels in Non-Transgenic Col-0 Lines. (DOCX)
