# Supplementary figures and images for: Genome-Wide Assessment of Efficiency and Specificity in CRISPR/Cas9 Mediated Multiple Site Targeting in Arabidopsis
Source: PLoS One. 2016 Sep 13;11(9):e0162169. doi: 10.1371/journal.pone.0162169 (PMC5021288; doi:10.1371/journal.pone.0162169)

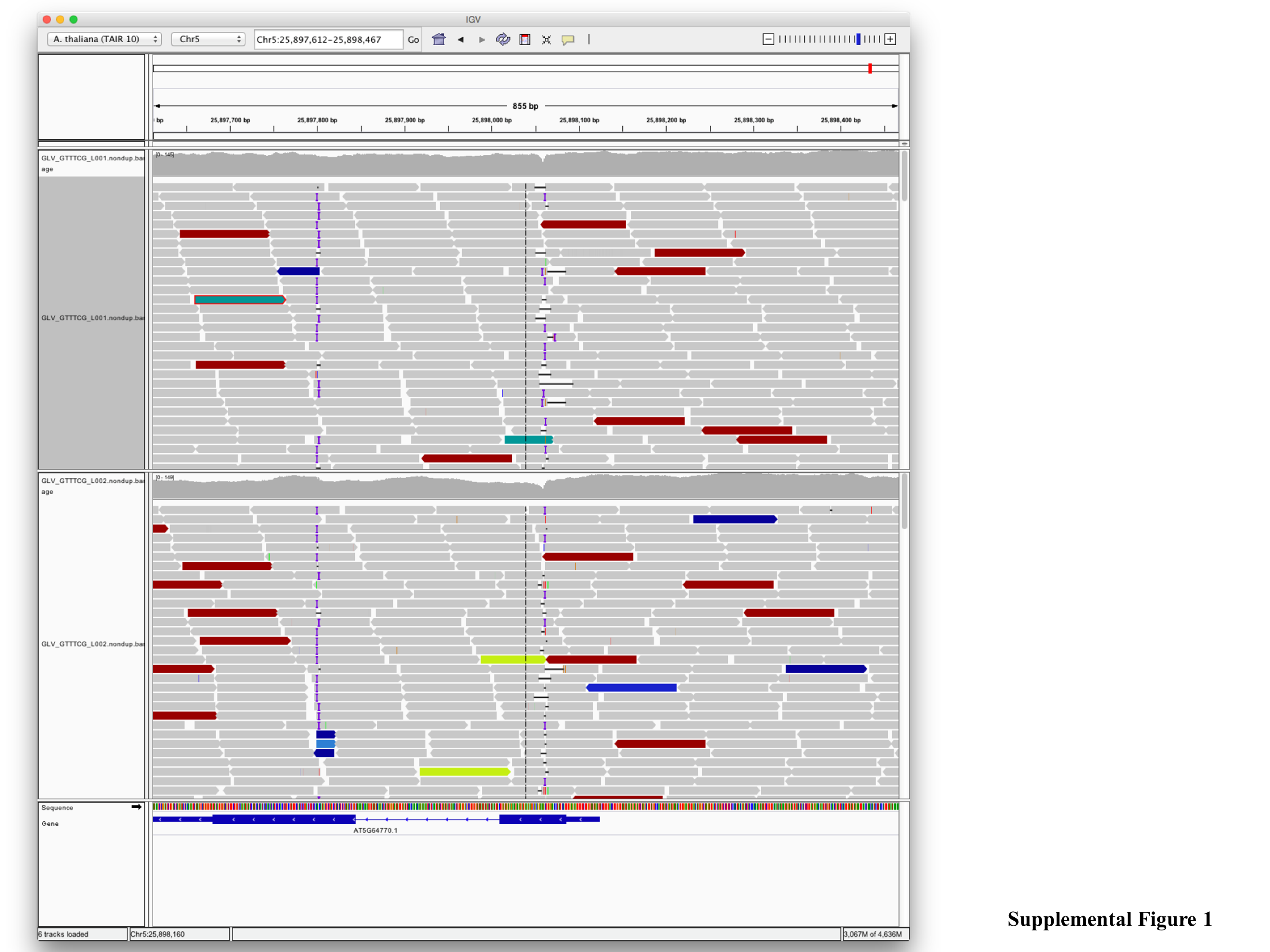

Supplement: S1 Fig — Sequencing lanes were kept separate and are displayed in different panels (upper panel is lane 1, lower panel is lane 2). Blue symbols represent insertions and dashes represent deletions in the mapped reads. In this view, the editing events can be seen for both site 1 and site 2. See https://www.broadinstitute.org/igv/ for a complete description and guide for the use of the IGV. (TIF) [file pone.0162169.s001.tif]

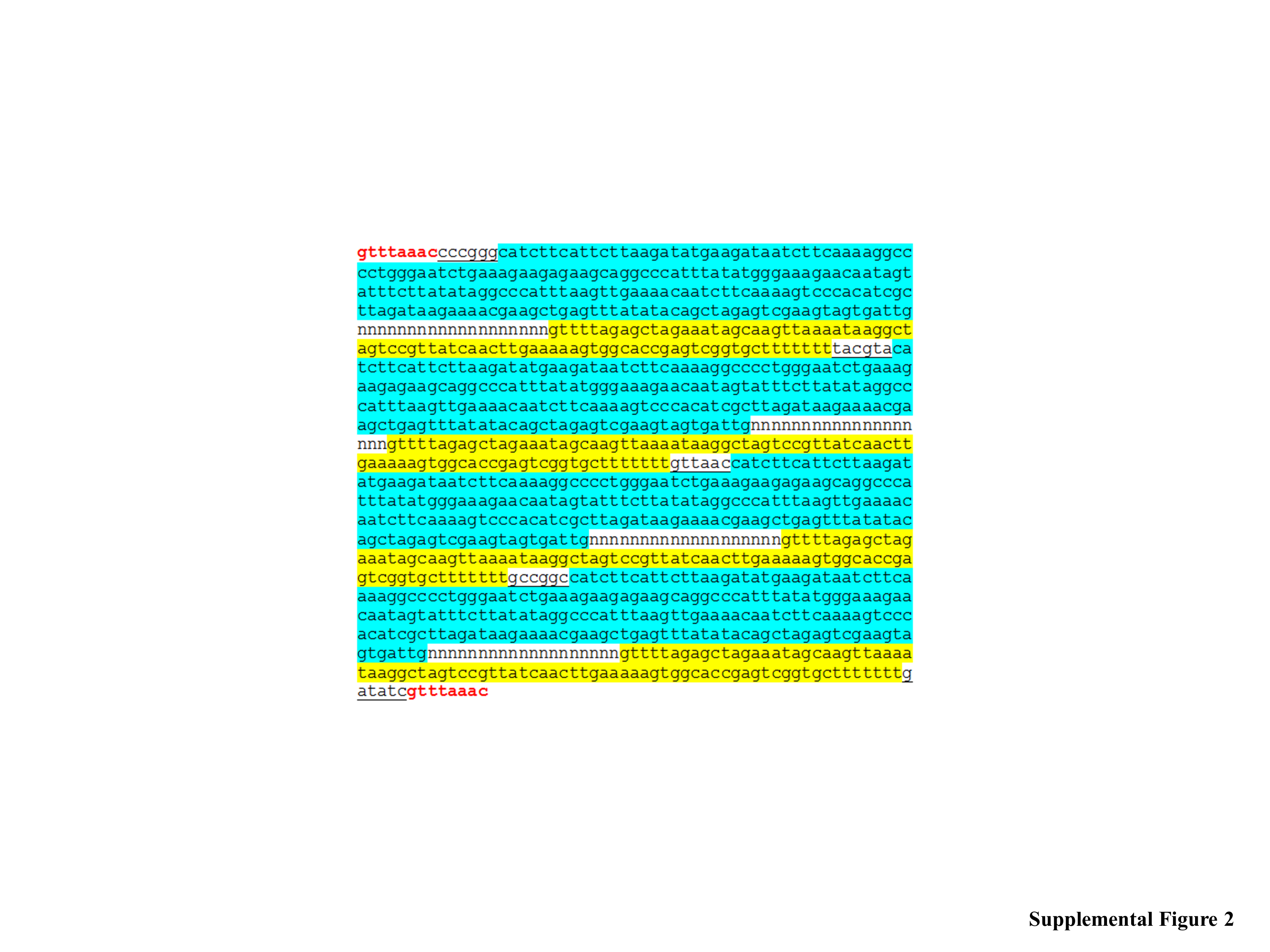

Supplement: S2 Fig — A 4 gRNA unit array. Each unit contains an Arabidopsis U6 promoter (blue), target site pasting location (poly n streach), and gRNA backbone with poly T terminator (yellow). Each unit is separated by a unique blunt restriction sites (underlined) in the following order: SmaI, SnaBI, HpaI, NaeI, EcoRV (S3 Fig). The entire array is flanked by PmeI sites on either site (Red). Higher order arrays can be synthesized but take considerably longer than a 4 unit array. Note: the poly n stretch is proceeded in each case by a transcriptional G. Each poly N stretch is replaced with the desired genomic target site, both 19 and 20 nt target site will work. (TIF) [file pone.0162169.s002.tif]

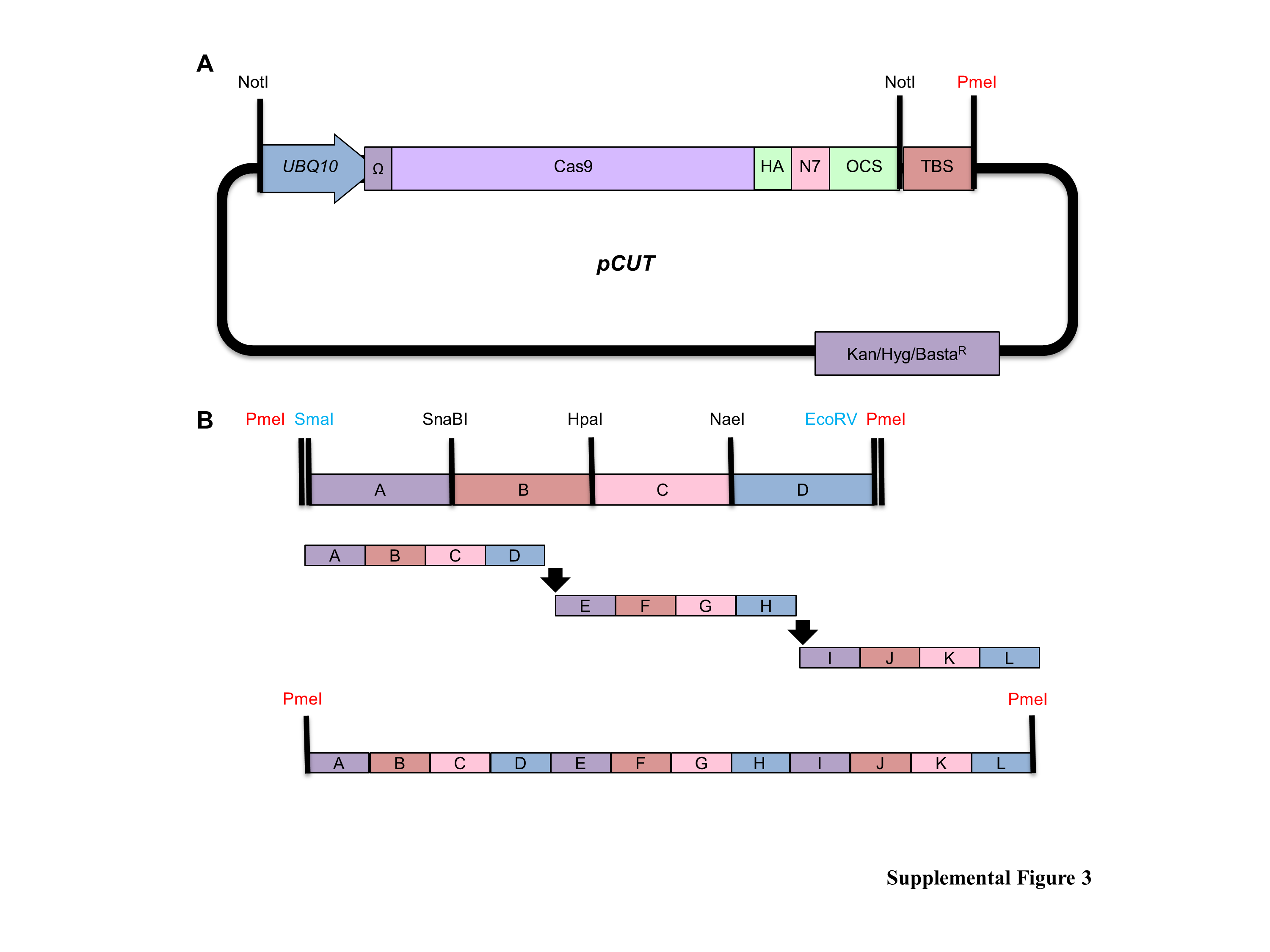

Supplement: S3 Fig — A) The pCUT binary vector system. A plant codon optimized HA-tagged nuclear localized (N7) Cas9 is expressed from the Arabidopsis UBQ10 promoter. A transformer boosting sequence (TBS [1]) separates the Cas9 expression cassette from a unique PmeI site (red). OCS; octopine synthase terminator. B) Strategy for array stacking. Array gRNA units (A-L), are synthesized or cloned in groups of four containing flanking PmeI sites (red) and unique blunt cutting sites (blue). Arrays are stacked as PmeI fragments cloned into unique Sma1 or EcoRV blunt sites iteratively to preserve the flanking PmeI sites. Example shows stacking of three 4 unit arrays to create a 12 unit stacked array. Reference 1: Hily JM, Singer SD, Yang Y, Liu Z. A transformation booster sequence (TBS) from Petunia hybrida functions as an enhancer-blocking insulator in Arabidopsis thaliana. Plant cell reports. 2009;28(7):1095–104. doi: 10.1007/s00299-009-0700-8. PubMed PMID: 19373469. (TIF) [file pone.0162169.s003.tif]
