# Supplementary material for: Genome-Wide Assessment of Efficiency and Specificity in CRISPR/Cas9 Mediated Multiple Site Targeting in Arabidopsis
Source: PLoS One. 2016 Sep 13;11(9):e0162169. doi: 10.1371/journal.pone.0162169 (PMC5021288; doi:10.1371/journal.pone.0162169)
Supplement: S1 Table — (DOCX) [file pone.0162169.s004.docx]

S1 Table. Genomic target sites for Cas9

| Gene | Guide | Guide Sequence |
| --- | --- | --- |
| CLE18 | 1 | tcgaggaaaacaatatcgac |
| CLE18 | 2 | aaaacaatatcgaccggtct |
| GLV1 | 1 | cttctttggacgtcttcaa |
| GLV1 | 2 | gagaaaggaggcgggcgtt |
| GLV2 | 1 | tgcgacgaggaaagacttg |
| GLV2 | 2 | aatagaacgctattggttg |
| GLV6 | 1 | gctagtgcacaaagaaaga |
| GLV6 | 2 | tggagttacaagcaataaaa |
| GLV7 | 1 | tgatgaaaaagatgatacg |
| GLV7 | 2 | ctgaaacgaagatgaagag |
| GLV8 | 1 | atcaatcccaaaaagaagaa |
| GLV8 | 2 | aagacaagcaataaagctg |
| GLV10 | 1 | aaggatcattgaagcaaca |
| GLV10 | 2 | gataatctgcaaataagag |

All genomic target sites listed 5’-3’.
