# Supplementary material for: Genome-Wide Assessment of Efficiency and Specificity in CRISPR/Cas9 Mediated Multiple Site Targeting in Arabidopsis
Source: PLoS One. 2016 Sep 13;11(9):e0162169. doi: 10.1371/journal.pone.0162169 (PMC5021288; doi:10.1371/journal.pone.0162169)
Supplement: S2 Table — (DOCX) [file pone.0162169.s005.docx]

**S2 Table. Primers used for PCR amplification and sequencing**

| Gene | Primer | Sequence |
| --- | --- | --- |
| CLE18 | CLE18 CR F | ctaatacaaactatatctgtatacgc |
| CLE18 | CLE18 CR R | ccaactaaaaaaatctatacaaaaac |
| GLV1 | GLV1 CR F | gaggagaaatagagaacaaagaag |
| GLV1 | GLV1 CR R | gtctcatctccctgtatacag |
| GLV2 | GLV2.2 CR F | gataatactgtacacatgcatacaaag |
| GLV2 | GLV2.2 CR R | gtgggaagacagagaaatatcgacg |
| GLV6 | GLV6 CR F | gtgtttcgtgcacttatctcacatag |
| GLV6 | GLV6 CR R | gaaatcaaactggagcttcaatatg |
| GLV7 | GLV7.2 CR F | gcttcaaaggttcaaaattttgg |
| GLV7 | GLV7.2 CR R | gagaaacaacataattgtaaatag |
| GLV8 | GLV8 CR F | ggatacagtataaaaacatcataatag |
| GLV8 | GLV8 CR R | gcactggtcattggacatccggtg |
| GLV10 | GLV10 CR F | gactcatgtttggcttttcccatgag |
| GLV10 | GLV10 CR R | gatagtttgagcagctatgaatgcg |
