# Supplementary material for: Genome-Wide Assessment of Efficiency and Specificity in CRISPR/Cas9 Mediated Multiple Site Targeting in Arabidopsis
Source: PLoS One. 2016 Sep 13;11(9):e0162169. doi: 10.1371/journal.pone.0162169 (PMC5021288; doi:10.1371/journal.pone.0162169)
Supplement: S3 Table — (DOCX) [file pone.0162169.s006.docx]

**S3 Table. On-Target Rates**

| **Site** | **Gene** | **Insertions** | **Deletions** | **Total Events^a^** | **Total Reads** | **Percent^b^** |
| --- | --- | --- | --- | --- | --- | --- |
| CLE18_1 | AT1G66145.1 | 97 | 16 | 113 | 217 | 52.1 |
| CLE18_2 | AT1G66145.1 | 0 | 0 | 0 | 0 | 0 |
| GLV1_1 | AT4G16515.1 | 23 | 101 | 124 | 181 | 68.5 |
| GLV1_2 | AT4G16515.1 | 93 | 28 | 121 | 183 | 66.1 |
| GLV2_1 | AT5G64770.1 | 50 | 36 | 86 | 231 | 37.2 |
| GLV2_2 | AT5G64770.1 | 90 | 31 | 121 | 219 | 55.3 |
| GLV6_1 | AT2G03830.1 | 168 | 51 | 219 | 251 | 87.3 |
| GLV6_2 | AT2G03830.1 | 87 | 3 | 90 | 267 | 33.7 |
| GLV7_1 | AT2G04025.1 | 65 | 25 | 90 | 271 | 33.2 |
| GLV7_2 | AT2G04025.1 | 111 | 38 | 149 | 198 | 75.3 |
| GLV8_1 | AT3G02242.1 | 0 | 0 | 0 | 0 | 0 |
| GLV8_2 | AT3G02242.1 | 127 | 58 | 185 | 243 | 76.1 |
| GLV10_1 | AT5G51451.1 | 28 | 92 | 167 | 250 | 66.8 |
| GLV10_2 | AT5G51451.1 | 231 | 8 | 239 | 259 | 92.3 |
| **Total** |  |  |  | 1704 | 2770 | 61.5 |

a) Total number of editing events, including both insertions and deletions

b) Number of editing events per number of reads analyzed
