# Supplementary material for: Genome-Wide Assessment of Efficiency and Specificity in CRISPR/Cas9 Mediated Multiple Site Targeting in Arabidopsis
Source: PLoS One. 2016 Sep 13;11(9):e0162169. doi: 10.1371/journal.pone.0162169 (PMC5021288; doi:10.1371/journal.pone.0162169)
Supplement: S4 Table — (DOCX) [file pone.0162169.s007.docx]

**S4 Table. Comparison of model fits for insertions, deletions and additional explanatory variables.**

| Model parameters | k | ΔAICc | logLik | Chisq | p-value |
| --- | --- | --- | --- | --- | --- |
| Insertions | 4 | 0 | -67.6 | 19.8793 | 0.00000031 |
| Insertions+site+distance from PAM | 9 | 6.7 | -65.9 | 3.338 | 0.648 |
| None | 3 | 19.7 | -78.4 |  |  |
| Deletions | 4 | 19.9 | -77.5 | 1.845 | 0.1743 |
