# Supplementary material for: Genome-Wide Assessment of Efficiency and Specificity in CRISPR/Cas9 Mediated Multiple Site Targeting in Arabidopsis
Source: PLoS One. 2016 Sep 13;11(9):e0162169. doi: 10.1371/journal.pone.0162169 (PMC5021288; doi:10.1371/journal.pone.0162169)
Supplement: S5 Table — (DOCX) [file pone.0162169.s008.docx]

**S5 Table. Off-Target Rates**

| **Site** | **Off-Target Sequence^a^** | **Score^b^** | **Mismatch^c^** | **Location** | **Gene** | **Mutation Type** | **Indels** | **SNPs** | **Reads** | **Percent Events^d^** | **Percent Editing^e^** |
| --- | --- | --- | --- | --- | --- | --- | --- | --- | --- | --- | --- |
| CLE18_2 | taaccaaaattgaccggtctagg | 0.8 | 4MMs | 3:-5546918 |  | Intergenic | 0 | 0 | 247 | 0 | 0 |
|  | taaacaatttaaaccggtcttag | 0.2 | 4MMs | 4:-9040312 | AT4G15940 | utr | 0 | 1 | 259 | 0 | 0 |
|  | caaagaatatcgacggttcttag | 0.2 | 4MMs | 3:-1287028 | AT3G04721 | exon | 0 | 1 | 221 | 0 | 0 |
|  | aaaacaatatgaagcggtctcgg | 0.1 | 3MMs | 1:-17073343 | AT1G45140 | utr | 0 | 1 | 291 | 0 | 0 |
|  | aaaacaatatgaagcggtctcgg | 0.1 | 3MMs | 3:+15350843 | AT3G43425 | utr | 0 | 0 | 250 | 0 | 0 |
|  | aaaacaatatgaagcggtttcgg | 0 | 4MMs | 4:-4143423 | AT4G07336 | utr | 0 | 0 | 265 | 0 | 0 |
| GLV1_1 | gcttcttaggccatcttcaacgg | 0.5 | 3MMs | 3:+5328561 |  | Intergenic | 0 | 0 | 258 | 0 | 0 |
|  | gcatgtttgaacgtctgcaaagg | 0.5 | 4MMs | 1:-5747240 | AT1G16800 | utr | 0 | 0 | 256 | 0 | 0 |
|  | gtctcttttgacgtctccaatgg | 0.4 | 4MMs | 1:-10078904 | AT1G28680 | utr | 0 | 0 | 227 | 0 | 0 |
|  | gtttctttttatgtcttcaatag | 0.4 | 4MMs | 5:+23271377 | AT5G57440 | utr | 0 | 2 | 248 | 0.8 | 0 |
|  | gcctttttggacttcttcatcgg | 0.3 | 4MMs | 4:+243237 | AT4G00570 | utr | 0 | 0 | 260 | 0.0 | 0 |
|  | ggttctttgctcgtcttcgacgg | 0.3 | 4MMs | 1:+29576019 | AT1G78630 | utr | 0 | 3 | 178 | 1.7 | 0 |
|  | gcttctttggatttcttcaccag | 0.2 | 3MMs | 5:-13887791 | AT5G35725 | utr | 0 | 0 | 263 | 0 | 0 |
|  | gcttttttggatatcttccaagg | 0.1 | 4MMs | 4:-14392181 | AT4G29190 | utr | 0 | 0 | 242 | 0 | 0 |
|  | gcttcttttgatttcttcactag | 0.1 | 4MMs | 1:+11917390 | AT1G32890 | utr | 0 | 0 | 232 | 0 | 0 |
|  | acttctttggttgtcatcaatag | 0.1 | 4MMs | 5:+7121633 | AT5G20960 | intron | 0 | 1 | 265 | 0 | 0 |
|  | gcttctctggagagcttcaatgg | 0 | 4MMs | 1:+25643205 | AT1G68390 | utr | 0 | 1 | 228 | 0 | 0 |
|  | gcttctttcgtcatctttaatag | 0 | 4MMs | 2:+14717421 |  | Intergenic | 0 | 0 | 229 | 0 | 0 |
|  | gcttccatggtcgccttcaaggg | 0 | 4MMs | 5:+4081933 | AT5G12920 | intron | 0 | 2 | 228 | 0 | 0 |
| GLV1_2 | ggagaagataggtgggcgttagg | 0.2 | 4MMs | 5:-19308921 |  | Intergenic | 0 | 9 | 204 | 4.4 | 0 |
|  | ggatacaggaagtgggcgttgag | 0.2 | 4MMs | 1:+14337003 | AT1G38185 | utr | 0 | 0 | 197 | 0 | 0 |
|  | ggagaactgaagccggcgttgag | 0.1 | 4MMs | 3:-20788776 | AT3G56010 | utr | 0 | 0 | 255 | 0 | 0 |
|  | ggagaatggaggcgtgggatgag | 0 | 4MMs | 3:-3043414 | AT3G09920 | utr | 0 | 0 | 209 | 0 | 0 |
|  | ggagaaaggagggttccgttgag | 0 | 4MMs | 4:-5315029 | AT4G08390 | utr | 0 | 1 | 205 | 0 | 0 |
| GLV2_1 | gggagacgaggagagacttggag | 1.1 | 3MMs | 3:-19697966 | AT3G53150 | exon | 0 | 0 | 255 | 0 | 0 |
|  | gtgcaacaaggaaagacgtgtgg | 0.6 | 3MMs | 3:+10736752 | AT3G28650 | exon | 0 | 0 | 250 | 0 | 0 |
|  | gtgcgacgaagtaagactcgcag | 0.4 | 3MMs | 2:-14611972 | AT2G34660 | utr | 0 | 0 | 230 | 0 | 0 |
|  | ttgagacgatgaaagccttgtgg | 0.3 | 4MMs | 4:-3156978 | AT4G06499 | utr | 0 | 0 | 253 | 0 | 0 |
|  | gggagatgaggaaatacttgagg | 0.3 | 4MMs | 5:-26380679 | AT5G65950 | intron | 0 | 0 | 167 | 0 | 0 |
|  | gtgcaacgaggaaagaagtgtgg | 0.2 | 3MMs | 2:+1569005 | AT2G04500 | utr | 0 | 0 | 255 | 0 | 0 |
|  | gtgcaactagaaaagacgtgtgg | 0.2 | 4MMs | 2:+17965018 | AT2G43220 | utr | 0 | 0 | 240 | 0 | 0 |
|  | gtgcaacgaggaatgacgtgtgg | 0.1 | 3MMs | 1:-20702552 | AT1G55440 | exon | 0 | 0 | 246 | 0 | 0 |
|  | gtgcgacaagggaagatgtgtgg | 0.1 | 4MMs | 5:+18552672 | AT5G45730 | utr | 0 | 1 | 212 | 0 | 0 |
|  | gtgcgacgaggaaagaccgaggg | 0.1 | 3MMs | 1:-22114101 | AT1G60030 | utr | 0 | 1 | 251 | 0 | 0 |
|  | gtgcaacaaggaaagacgtatgg | 0.1 | 4MMs | 3:-16702313 | AT3G45530 | utr | 0 | 1 | 260 | 0 | 0 |
|  | gtgcaacaaggaaagatgtgtgg | 0.1 | 4MMs | 3:-9993089 |  | Intergenic | 0 | 0 | 247 | 0 | 0 |
|  | gtccgaagaggaaggatttgtag | 0.1 | 4MMs | 5:-25450652 | AT5G63560 | utr | 0 | 1 | 208 | 0 | 0 |
|  | gtgcaacaaggaatgacgtgtgg | 0 | 4MMs | 1:-20677675 | AT1G55380 | exon | 0 | 7 | 206 | 3.4 | 0 |
|  | gtgcaacaaggaatgacgtgtgg | 0 | 4MMs | 1:-20692903 | AT1G55420 | exon | 0 | 9 | 208 | 4.3 | 0 |
|  | gtgcgacgaggaaaagttttggg | 0 | 4MMs | 4:+17413916 |  | Intergenic | 0 | 0 | 254 | 0 | 0 |
|  | gtgccacgtggaaaagcttgagg | 0 | 4MMs | 4:+14950133 |  | Intergenic | 0 | 0 | 265 | 0 | 0 |
| GLV2_2 | No off targets identified |  |  |  |  |  |  |  |  |  |  |
| GLV6_1 | agatggtgcacagagaaagaagg | 0.6 | 4MMs | 3:-6690498 | AT3G19300 | utr | 0 | 0 | 198 | 0 | 0 |
|  | ggctaggggactaagaaagagag | 0.5 | 3MMs | 5:-6950561 | AT5G20540 | intron | 0 | 0 | 227 | 0 | 0 |
|  | aggtagtgaagaaagaaagagag | 0.5 | 4MMs | 3:-613851 | AT3G02830 | utr | 0 | 1 | 260 | 0 | 0 |
|  | agttggtgcacaaaaaaagagag | 0.4 | 4MMs | 5:-20377658 |  | Intergenic | 0 | 0 | 284 | 0 | 0 |
|  | gactattgtaaaaagaaagatgg | 0.3 | 4MMs | 2:-7414695 | AT2G17050 | utr | 0 | 1 | 230 | 0 | 0 |
|  | ggcgggtgtacaaagaaaaagag | 0.3 | 4MMs | 1:-3816222 | AT1G11340 | utr | 0 | 0 | 223 | 0 | 0 |
|  | ggctagtgaacaaaagaagaggg | 0.1 | 3MMs | 1:+10230162 | AT1G29270 | utr | 0 | 1 | 246 | 0 | 0 |
|  | gagtagtgcataatgaaagagag | 0.1 | 4MMs | 3:-16632186 |  | Intergenic | 0 | 0 | 256 | 0 | 0 |
|  | ggttggtgcacaaaaaaaaaagg | 0.1 | 4MMs | 1:-28498701 |  | Intergenic | 0 | 1 | 251 | 0 | 0 |
| GLV6_2 | ttgagttaaaagaaataaaagag | 0.7 | 3MMs | 1:+13964473 |  | Intergenic | 0 | 1 | 291 | 0 | 0 |
|  | tggatttataaggaataaaacgg | 0.6 | 3MMs | 4:-12587649 |  | Intergenic | 0 | 0 | 262 | 0 | 0 |
|  | agaagataaaagcaataaaatag | 0.5 | 4MMs | 1:-12218432 |  | Intergenic | 0 | 0 | 226 | 0 | 0 |
|  | aggagaaagaagcaataaaaagg | 0.4 | 4MMs | 3:+20645732 | AT3G55646 | utr | 0 | 0 | 269 | 0 | 0 |
|  | tgaagttaccatcaatgaaatgg | 0.3 | 4MMs | 3:+7073061 | AT3G20280 | utr | 0 | 0 | 242 | 0 | 0 |
|  | aacagttacaagcaaaaaaaagg | 0.3 | 4MMs | 5:-26430340 | AT5G66110 | intron | 0 | 1 | 253 | 0 | 0 |
|  | ttggtttacaagcaagaaaatag | 0.3 | 4MMs | 5:-14690377 |  | Intergenic | 0 | 0 | 280 | 0 | 0 |
|  | cgaagttacaaagaataaaatag | 0.3 | 4MMs | 1:-2851050 | AT1G08890 | intron | 0 | 0 | 218 | 0 | 0 |
|  | tgtggttacaagaaataaattag | 0.3 | 4MMs | 1:-16931521 |  | Intergenic | 0 | 0 | 260 | 0 | 0 |
|  | tgtagtttcatggaataaaaagg | 0.3 | 4MMs | 4:+17141356 |  | Intergenic | 0 | 1 | 256 | 0 | 0 |
|  | atgaggtacaagcaaaaaaatgg | 0.2 | 4MMs | 5:-15811738 |  | Intergenic | 0 | 0 | 252 | 0 | 0 |
|  | ctgagttaaaagcaaaaaaaagg | 0.2 | 4MMs | 3:-8109324 |  | Intergenic | 0 | 1 | 247 | 0 | 0 |
|  | tggagatacaagtaatgaattag | 0.1 | 4MMs | 3:-11529633 |  | Intergenic | 0 | 0 | 248 | 0 | 0 |
|  | tggagaaacaaaaaataaaatag | 0.1 | 4MMs | 4:-15808020 |  | Intergenic | 0 | 1 | 290 | 0 | 0 |
|  | tgaagttgcaagcaaaagaaggg | 0.1 | 4MMs | 4:-5795165 | AT4G09040 | utr | 0 | 0 | 241 | 0 | 0 |
|  | tggagccaaaagcaataataagg | 0.1 | 4MMs | 5:+25503992 |  | Intergenic | 0 | 1 | 335 | 0 | 0 |
|  | tggagtgaaaaacaattaaaggg | 0.1 | 4MMs | 1:+23337312 |  | Intergenic | 0 | 1 | 249 | 0 | 0 |
|  | tggagttaaaagcgagaaatcgg | 0 | 4MMs | 5:-23322084 | AT5G57590 | utr | 0 | 1 | 223 | 0 | 0 |
|  | tggagttaaaagcaaggcaaggg | 0 | 4MMs | 1:-24513246 | AT1G65890 | utr | 0 | 1 | 276 | 0 | 0 |
|  | tggagttagaggcaatattaagg | 0 | 4MMs | 3:-20936751 | AT3G56480 | | 0 | 0 | 241 | 0 | 0 |
| GLV7_1 | gtgtgaagaaagatgatacgaag | 0.8 | 4MMs | 1:-9397993 | AT1G27070 | utr | 0 | 0 | 281 | **0** | 0 |
|  | gagttgaaagagatgatacaagg | 0.6 | 4MMs | 5:-17703222 | AT5G44000 | utr | 0 | 0 | 212 | 0 | 0 |
|  | atgatgatgaagatgatactggg | 0.4 | 4MMs | 2:+10022823 | AT2G23530 | utr | 0 | 2 | 247 | 0.8 | 0 |
|  | gagatgaaaaacatgattcgaag | 0.3 | 3MMs | 5:-17085133 |  | Intergenic | 0 | 1 | 261 | 0.0 | 0 |
|  | ctgatgagaaacatgatacatgg | 0.3 | 4MMs | 3:-7916423 | AT3G22380 | intron | 0 | 0 | 214 | 0.0 | 0 |
|  | ttgaggaagaagatgataagaag | 0.3 | 4MMs | 4:+18504970 | AT4G39880 | utr | 0 | 0 | 249 | 0.0 | 0 |
|  | ttgaggaagaagatgataagaag | 0.3 | 4MMs | 5:+2829163 |  | Intergenic | 0 | 0 | 235 | 0.0 | 0 |
|  | gtgctgaaatcgatgaaacgaag | 0.3 | 4MMs | 3:+10682312 | AT3G28500 | utr | 0 | 0 | 241 | 0.0 | 0 |
|  | gtaatgcaaaaaatgaaacgagg | 0.2 | 4MMs | 4:-15206755 | AT4G31340 | intron | 0 | 0 | 245 | 0.0 | 0 |
|  | atgatgaatcagatgatgcgaag | 0.2 | 4MMs | 2:-19296791 | AT2G46970 | utr | 0 | 0 | 266 | 0.0 | 0 |
|  | gtgatggcaaacatgatacaaag | 0.2 | 4MMs | 5:-5687742 | AT5G17290 | intron | 0 | 0 | 290 | 0.0 | 0 |
|  | ttgatgaagaaaatgataagaag | 0.2 | 4MMs | 2:+4654480 |  | Intergenic | 0 | 0 | 288 | 0.0 | 0 |
|  | gtgatgaaaaagttgatgagaag | 0.1 | 3MMs | 1:-6897601 | AT1G19870 | utr | 0 | 0 | 259 | 0.0 | 0 |
|  | gcgatgaaaaaaatgagaccagg | 0.1 | 4MMs | 5:-19255190 | AT5G47470 | utr | 0 | 1 | 252 | 0.0 | 0 |
|  | gagaagaaaaagaagagacgagg | 0.1 | 4MMs | 3:-687331 | AT3G03050 | utr | 0 | 0 | 233 | 0.0 | 0 |
|  | gtgaagtaaaagatgctaagagg | 0.1 | 4MMs | 2:+860283 | AT2G02955 | utr | 0 | 0 | 264 | 0.0 | 0 |
|  | gagatgaagaagatgagaggagg | 0.1 | 4MMs | 5:+6014664 | AT5G18190 | utr | 0 | 3 | 236 | 1.3 | 0 |
|  | gagatgaagaagatgagaggagg | 0.1 | 4MMs | 1:+12021244 |  | Intergenic | 0 | 3 | 215 | 1.4 | 0 |
|  | gtcattaaaaggattatacgggg | 0.1 | 4MMs | 5:-6685030 |  | Intergenic | 0 | 0 | 276 | 0 | 0 |
|  | gtgatggagaagatgataaatgg | 0.1 | 4MMs | 2:-10831256 | AT2G25450 | utr | 0 | 0 | 215 | 0 | 0 |
| GLV7_2 | gaagaaatgaagatgaagaggag | 2.5 | 3MMs | 4:-13017289 |  | Intergenic | 0 | 1 | 241 | 0 | 0 |
|  | actgaaatgaagatgaagaagag | 1.5 | 3MMs | 1:-2096464 |  | Intergenic | 0 | 0 | 219 | 0 | 0 |
|  | gttgatactaagatgaagagagg | 1 | 3MMs | 5:-6078400 | AT5G18350 | utr | 0 | 0 | 267 | 0 | 0 |
|  | gttgaatcgaagatgaagaagag | 1 | 3MMs | 3:-20173255 | AT3G54480 | utr | 0 | 0 | 246 | 0 | 0 |
|  | tctcaaacgaagatcaagagaag | 0.8 | 3MMs | 5:-19959117 | AT5G49230 | utr | 0 | 0 | 266 | 0 | 0 |
|  | cgagaaacaaagatgaagagaag | 0.8 | 4MMs | 3:+19346424 | AT3G52160 | utr | 0 | 1 | 255 | 0 | 0 |
|  | gcaaaaaagaagctgaagagaag | 0.6 | 4MMs | 4:+16867859 | AT4G35520 | utr | 0 | 0 | 218 | 0 | 0 |
|  | gctagaacaacgatgaagagaag | 0.5 | 4MMs | 1:-24812454 | AT1G66500 | utr | 0 | 0 | 197 | 0 | 0 |
|  | gcgaaaaggaagatgaagcgaag | 0.5 | 4MMs | 2:+16529505 |  | Intergenic | 0 | 0 | 236 | 0 | 0 |
|  | gacgaaacgaagaagaagagaag | 0.4 | 3MMs | 1:+26980900 | AT1G71710 | utr | 0 | 0 | 265 | 0 | 0 |
|  | gcgaaaacgactatgaagagaag | 0.4 | 4MMs | 1:-22606151 | AT1G61290 | utr | 0 | 0 | 224 | 0 | 0 |
|  | gctgaagggtagatgaagaaaag | 0.4 | 4MMs | 2:+4480295 | AT2G11240 | utr | 0 | 0 | 239 | 0 | 0 |
|  | gctaacaggaagctgaagagtgg | 0.3 | 4MMs | 3:-7141061 | AT3G20470 | utr | 0 | 0 | 218 | 0 | 0 |
|  | gataaaacaaagatcaagagaag | 0.3 | 4MMs | 3:+2145016 | AT3G06790 | intron | 0 | 0 | 251 | 0 | 0 |
|  | gttcaaaagaagaagaagagagg | 0.2 | 4MMs | 1:-15917131 | AT1G42460 | utr | 0 | 0 | 220 | 0 | 0 |
|  | gcggaaacacagataaagagaag | 0.2 | 4MMs | 2:-3523627 | AT2G07690 | utr | 0 | 0 | 233 | 0 | 0 |
|  | gttgcatcgaagaagaagagaag | 0.2 | 4MMs | 1:-22690775 | AT1G61500 | utr | 0 | 0 | 234 | 0 | 0 |
|  | ggagaaaggaagaagaagagaag | 0.2 | 4MMs | 1:-9271003 |  | Intergenic | 0 | 0 | 250 | 0 | 0 |
|  | gaagaaatgaagaggaagagaag | 0.2 | 4MMs | 2:-6546409 |  | Intergenic | 0 | 1 | 253 | 0 | 0 |
|  | gctgataagaacctgaagagaag | 0.2 | 4MMs | 1:-27135668 | AT1G72125 | utr | 0 | 2 | 234 | 0 | 0 |
| GLV8_1 | aactataccaaaaagaagaaaag | 1.7 | 3MMs | 2:+16826306 | AT2G40280 | intron | 0 | 1 | 213 | 0 | 0 |
|  | atgaatagcaaaaagaagaagag | 1.7 | 3MMs | 4:-285018 | AT4G00695 | utr | 0 | 0 | 248 | 0 | 0 |
|  | atcactgtcaaaaagaagaagag | 1.6 | 3MMs | 5:-26874607 |  | Intergenic | 0 | 0 | 239 | 0 | 0 |
|  | agcacacccaaaaagaagaaaag | 1.5 | 3MMs | 2:+18408489 |  | Intergenic | 0 | 0 | 257 | 0 | 0 |
|  | atcaaccccaaaaagaaaaaggg | 1.2 | 2MMs | 1:-329288 | AT1G01950 | intron | 0 | 1 | 265 | 0 | 0 |
|  | atcaattcgcaaaagaagaaaag | 0.9 | 3MMs | 1:+29195837 | AT1G77680 | intron | 0 | 1 | 245 | 0 | 0 |
|  | atgaaaccaaaaaagaagaagag | 0.9 | 3MMs | 3:-5546999 |  | Intergenic | 0 | 0 | 238 | 0 | 0 |
|  | aacaaccccaagaagaagaacag | 0.8 | 3MMs | 5:-12550627 | AT5G33280 | utr | 0 | 2 | 213 | 0 | 0 |
|  | aaacagcccaaaaagaagaacag | 0.8 | 4MMs | 3:-15365375 |  | Intergenic | 0 | 1 | 226 | 0 | 0 |
|  | atcaattcccaaaagaagaggag | 0.8 | 3MMs | 3:-14917084 | AT3G42806 | utr | 0 | 1 | 262 | 0 | 0 |
|  | attaatccgaagaagaagaagag | 0.8 | 3MMs | 1:-20142449 | AT1G53935 | intron | 0 | 0 | 256 | 0 | 0 |
|  | aagaatcacaagaagaagaatgg | 0.7 | 4MMs | 5:+4886497 | AT5G15080 | utr | 0 | 0 | 210 | 0 | 0 |
|  | agccttcccaagaagaagaaagg | 0.7 | 4MMs | 2:-4994709 | AT2G12385 | utr | 0 | 0 | 233 | 0 | 0 |
|  | atgaaacccaaatagaagaatag | 0.6 | 3MMs | 1:+20373782 | AT1G54560 | intron | 0 | 0 | 254 | 0 | 0 |
|  | atcattatcaagaagaagaaagg | 0.5 | 4MMs | 5:+6731599 | AT5G19910 | intron | 0 | 0 | 252 | 0 | 0 |
|  | atgaataacaaaaagaagattgg | 0.5 | 4MMs | 1:+30393524 | AT1G80870 | utr | 0 | 1 | 252 | 0 | 0 |
|  | ttcagccccacaaagaagaaagg | 0.5 | 4MMs | 1:-5780537 | AT1G16900 | utr | 0 | 0 | 268 | 0 | 0 |
|  | atcaagagccaaaagaagaacag | 0.5 | 4MMs | 1:-18583354 | AT1G50170 | intron | 0 | 1 | 240 | 0 | 0 |
|  | aaacatcccaaaaaaaagaaagg | 0.4 | 4MMs | 2:+5922512 | AT2G14070 | intron | 0 | 0 | 274 | 0 | 0 |
|  | aacaattccagtaagaagaacag | 0.3 | 4MMs | 1:+27093467 | AT1G71970 | | 0 | 0 | 280 | 0 | 0 |
| GLV8_2 | gaacagaagcaataaagctcaag | 0.8 | 3MMs | 4:-16162245 | AT4G33650 | intron | 0 | 4 | 293 | 1.4 | 0 |
|  | gaaaaaattcaataaagctgaag | 0.5 | 4MMs | 4:+17031541 | AT4G35980 | intron | 0 | 0 | 247 | 0.0 | 0 |
|  | gaaaacaaacaatgaagctgaag | 0.3 | 3MMs | 1:-5212925 | AT1G15150 | utr | 0 | 0 | 214 | 0.0 | 0 |
|  | gatgaaaagaaagaaagctgaag | 0.3 | 4MMs | 5:+16797664 | AT5G41990 | utr | 0 | 0 | 258 | 0.0 | 0 |
|  |  |  |  |  | AT5G41992 | | 0 | 0 | 0 | 0.0 | 0 |
|  | gaagcctaccaatagagctgagg | 0.2 | 4MMs | 4:+7054488 | AT4G11680 | utr | 0 | 0 | 249 | 0.0 | 0 |
|  | taaaaaaagcaataaagatgaag | 0.2 | 4MMs | 1:-5465329 |  | Intergenic | 0 | 2 | 235 | 0.9 | 0 |
|  | gaatacaagtactatagctgtgg | 0.2 | 4MMs | 5:-21173214 | AT5G52110 | utr | 0 | 0 | 251 | 0 | 0 |
|  | aaagaaaagcaataaaactcaag | 0.2 | 4MMs | 4:+5345972 |  | Intergenic | 0 | 0 | 230 | 0 | 0 |
|  | gatgacaagaaaaaaaactgcgg | 0.2 | 4MMs | 2:+11297394 |  | Intergenic | 0 | 2 | 207 | 0 | 0 |
|  | caagacaagaaaaaaaactgaag | 0.2 | 4MMs | 1:+22083488 | AT1G59980 | utr | 0 | 0 | 273 | 0 | 0 |
|  | gaagagaaacaacaaagctaagg | 0.1 | 4MMs | 3:-418585 | AT3G02240 | utr | 0 | 0 | 245 | 0 | 0 |
|  | ggaaacaaacaatgaagctgaag | 0.1 | 4MMs | 1:-5215726 | AT1G15160 | utr | 0 | 0 | 220 | 0 | 0 |
|  | gaagtcaagcaaaaaaactttgg | 0.1 | 4MMs | 2:-5163069 |  | Intergenic | 0 | 0 | 254 | 0 | 0 |
|  | gaagacaaggagaaaatctgtgg | 0.1 | 4MMs | 3:-19827831 | AT3G53480 | intron | 0 | 0 | 267 | 0 | 0 |
|  | aaagaaaagcaatataggtgaag | 0.1 | 4MMs | 1:+15851656 |  | Intergenic | 0 | 0 | 274 | 0 | 0 |
|  | aaagaaaagcaataaaggtcaag | 0.1 | 4MMs | 1:+15847571 |  | Intergenic | 0 | 0 | 246 | 0 | 0 |
|  | aaagacaagtaacaaagatgaag | 0.1 | 4MMs | 4:+12329322 | AT4G23660 | utr | 0 | 0 | 249 | 0 | 0 |
|  | caagaaaagcaataacgatgaag | 0 | 4MMs | 2:-10811416 |  | Intergenic | 0 | 0 | 246 | 0 | 0 |
|  | gcagataagcaatgaagcagaag | 0 | 4MMs | 2:-10451274 |  | Intergenic | 0 | 0 | 245 | 0 | 0 |
|  | gaagacaaaaaatacagatgtgg | 0 | 4MMs | 1:+22846811 |  | Intergenic | 0 | 0 | 273 | 0 | 0 |
| GLV10_1 | aaaagattattgaagcaacaagg | 2.6 | 3MMs | 1:-12405870 | AT1G34080 | utr | 0 | 0 | 225 | 0 | 0 |
|  | gaagaagcacagaagcaacaagg | 0.5 | 4MMs | 1:-24185982 | AT1G65110 | utr | 0 | 1 | 254 | 0 | 0 |
|  | gaagaaaaatagaagcaacaagg | 0.5 | 4MMs | 1:-24193469 | AT1G65120 | utr | 0 | 0 | 249 | 0 | 0 |
|  | gaagtatcattaaaacaacaggg | 0.4 | 3MMs | 2:-15338701 |  | Intergenic | 0 | 0 | 279 | 0 | 0 |
|  | gaagaatcgaagaagcaacatgg | 0.4 | 4MMs | 3:+8490893 |  | Intergenic | 0 | 1 | 239 | 0 | 0 |
|  | gaggcatcactgaaacaacaagg | 0.4 | 4MMs | 3:+22421954 | AT3G60660 | utr | 0 | 0 | 237 | 0 | 0 |
|  | caagattcattgaaacaacatgg | 0.3 | 4MMs | 2:-7953004 | AT2G18300 | utr | 0 | 0 | 225 | 0 | 0 |
|  | gaagaacaattgaagcaagaagg | 0.3 | 4MMs | 5:+8310212 | AT5G24350 | utr | 0 | 0 | 258 | 0 | 0 |
|  | gaagcagccttgaagcaacttag | 0.3 | 4MMs | 3:+1260217 | AT3G04630 | utr | 0 | 0 | 227 | 0 | 0 |
|  | caaggatcacggaaacaacatag | 0.2 | 4MMs | 2:-11635244 | AT2G27210 | intron | 0 | 0 | 213 | 0 | 0 |
|  | gaaagatgattgaagcaaatagg | 0.2 | 4MMs | 4:-13717507 |  | Intergenic | 0 | 0 | 261 | 0 | 0 |
|  | caaggatcatcaaagccacaggg | 0.2 | 4MMs | 5:-7209538 | AT5G21222 | utr | 0 | 0 | 218 | 0 | 0 |
|  | gaaggttcacagatgcaacatgg | 0.1 | 4MMs | 1:-12920761 |  | Intergenic | 0 | 0 | 245 | 0 | 0 |
|  | gaaggataattcatggaacatgg | 0 | 4MMs | 2:+4671783 | AT2G11640 | utr | 0 | 1 | 256 | 0 | 0 |
|  | gaaggatcatcgacggaaccagg | 0 | 4MMs | 4:-9150496 | AT4G16150 | utr | 0 | 0 | 253 | 0 | 0 |
|  | gaaagatcattggaacatcatag | 0 | 4MMs | 3:+11350101 |  | Intergenic | 0 | 1 | 176 | 0 | 0 |
| GLV10_2 | tgaacctctgcaaataagagggg | 0.8 | 4MMs | 3:+7701423 | AT3G21865 | intron | 0 | 0 | 240 | 0 | 0 |
|  | ggttaatctgaaaatatgagagg | 0.7 | 3MMs | 4:-17399080 |  | Intergenic | 0 | 0 | 317 | 0 | 0 |
|  | ccataatctctaaataagagcag | 0.7 | 4MMs | 1:+20406237 | AT1G54650 | utr | 0 | 0 | 241 | 0 | 0 |
|  | tgaaattctgcaaataagatggg | 0.4 | 4MMs | 2:+6645305 |  | Intergenic | 0 | 0 | 247 | 0 | 0 |
|  | ggatagtctccaaataaaagagg | 0.3 | 3MMs | 1:-22157069 | AT1G60090 | utr | 0 | 1 | 271 | 0 | 0 |
|  | gaataatttacaaatcagagagg | 0.2 | 4MMs | 1:+159907 | AT1G01440 | utr | 0 | 0 | 252 | 0 | 0 |
|  | caataatctgccagtaagagtgg | 0.1 | 4MMs | 5:-16703223 | AT5G41755 | utr | 0 | 1 | 205 | 0 | 0 |
|  | ggacaatctgttaaaaagagcag | 0.1 | 4MMs | 1:-838390 | AT1G03380 | intron | 0 | 0 | 235 | 0 | 0 |
|  | ggatagtctccaaatataagagg | 0.1 | 4MMs | 1:+22219901 | AT1G60260 | utr | 0 | 1 | 240 | 0 | 0 |
|  | ggattatctgcaaataccatggg | 0 | 4MMs | 4:-28282 | AT4G00060 | utr | 0 | 0 | 234 | 0 | 0 |
|  | ggataatcttcaaaccggagagg | 0 | 4MMs | 4:-8128603 | AT4G14103 | utr | 0 | 1 | 255 | 0 | 0 |
|  | ggataatcaacaaagaacagcag | 0 | 4MMs | 1:-5737976 | AT1G16770 | utr | 0 | 1 | 242 | 0 | 0 |
| **Total** |  |  |  |  |  |  |  |  | 43498 |  |  |

a) Possible off-target sites generated using CRISPR-P

b) Off-target potential calculated using CRISPR-P

c) Number of mismatches of off-target sequence compared to the original target site

d) Number of indels and SNPs combined per number of reads analyzed?

e) Number of indels per number of reads analyzed.
