# Supplementary material for: Genome-Wide Assessment of Efficiency and Specificity in CRISPR/Cas9 Mediated Multiple Site Targeting in Arabidopsis
Source: PLoS One. 2016 Sep 13;11(9):e0162169. doi: 10.1371/journal.pone.0162169 (PMC5021288; doi:10.1371/journal.pone.0162169)
Supplement: S6 Table — (DOCX) [file pone.0162169.s009.docx]

**S6 Table. pCUT binary vectors**

|  | Bacterial Selection | *in planta* Selection | Cloning Site |
| --- | --- | --- | --- |
| pCUT3 | spectinomycin | Kanamycin | PmeI |
| pCUT4 | spectinomycin | Hygromycin | PmeI |
| pCUT6 | spectinomycin | Basta | PmeI |
| pCUT3G | spectinomycin | Kanamycin | Gateway |
| pCUT4G | spectinomycin | Hygromycin | Gateway |
| pCUT6G | spectinomycin | Basta | Gateway |
