# Supplementary material for: Genome-Wide Assessment of Efficiency and Specificity in CRISPR/Cas9 Mediated Multiple Site Targeting in Arabidopsis
Source: PLoS One. 2016 Sep 13;11(9):e0162169. doi: 10.1371/journal.pone.0162169 (PMC5021288; doi:10.1371/journal.pone.0162169)
Supplement: S7 Table — (DOCX) [file pone.0162169.s010.docx]

**S7 Table. Indels in Non-Transgenic Col-0 Lines**

| **Target** | **Chr^a^** | **Sample** | **Sequence** | **Freq^b^** |
| --- | --- | --- | --- | --- |
| **1** | **1** | **Reference** | **CCAAAATGAAGAGAATTTTTTAAAACCCACATCCGGCCGGTTCAACCGATTCACCGGGTCATGGATTAATG** |  |
|  |  | **Variant** | **CCAAAATGAAGAGAATTTTTTAAAACCCACATCCGGCCGGTTCA---------CCGGTTCATGGATTAATG** | **0.57** |
|  |  | **Sample 1** | **CCAAAATGAAGAGAATTTTTTAAAACCCACATCCGGCCGGT-----------------TCATGGATTAATG** |  |
|  |  | **Sample 2** | **CCAAAATGAAGAGAATTTTTTAAAACCCACATCCGGCCGGT-----------------TCATGGATTAATG** |  |
|  |  | **Sample 3** | **CCAAAATGAAGAGAATTTTTTAAAACCCACATCCGGCCGGTTCAACCGATTCACCGGGTCATGGATTAATG** |  |
|  |  | **Sample 4** | **CCAAAATGAAGAGAATTTTTTAAAACCCACATCCGGCCGGTTCAACCGATTCACCGGGTCATGGATTAATG** |  |
|  |  | **Sample 5** | **CCAAAATGAAGAGAATTTTTTAAAACCCACATCCGGCCGGTTCAACCGATTCACCGGGTCATGGATTAATG** |  |
|  |  | **Sample 6** | **CCAAAATGAAGAGAATTTTTTAAAACCCACATCCGGCCGGTTCAACCGATTCACCGGGTCATGGATTAATG** |  |
| **2** | **3** | **Reference** | **TAGATTAGACCCATTCTCTAGATCTCAACAGGTTGTTCCTCC---TTCTTCCTATTTAGTTACTTGGTTTCAAT** |  |
|  |  | **Variant** | **TAGATTAGACCCATTCTCTAGATCTCAACAGGTTGTTCCTCCTTCTTCTTCCTATTTAGTTACTTGGTTTCAAT** | **0.67** |
|  |  | **Sample 1** | **TAGATTAGACCCATTCTCTA…overlapping traces** |  |
|  |  | **Sample 2** | **TAGATTAGACCCATTCTCTAGATC…overlapping traces** |  |
|  |  | **Sample 3** | **TAGATTAGACCCATTCTCTAGA…overlapping traces** |  |
|  |  | **Sample 4** | **TAGATTAGACCCATTCTCTA…overlapping traces** |  |
|  |  | **Sample 5** | **TAGATTAGACCCATTCTCTAGATC…overlapping traces** |  |
|  |  | **Sample 6** | **TAGATTAGACCCATTCTCTAGATCTCAACAGGTTGTTCCTCCTTCTTCTTCCTATTTAGTTACTTGGTTTCAAT** |  |
| **3** | **3** | **Reference** | **TGTAAAAACTTTATCGATACAAAAATCATTGCAAAAGGCAAAGCATGCAAGCTTCTTTTCCACATTTTTA** |  |
|  |  | **Variant** | **TGTAAAAACTTTATCGATACAAAAATCATTGCAAAAGGCAG-------AAGCTTCTTTTCCACATTTTTA** | **0.78** |
|  |  | **Sample 1** | **TGTAAAAACTTTATCGATACAAAAATCATTGCAAAAGGCAG-------AAGCTTCTTTTCCACATTTTTA** |  |
|  |  | **Sample 2** | **TGTAAAAACTTTATCGATACAAAAATCATTGCAAAAGGCAG-------AAGCTTCTTTTCCACATTTTTA** |  |
|  |  | **Sample 3** | **TGTAAAAACTTTATCGATACAAAAATCATTGCAAAAGGCAG-------AAGCTTCTTTTCCACATTTTTA** |  |
|  |  | **Sample 4** | **TGTAAAAACTTTATCGATACAAAAATCATTGCAAAAGGCAG-------AAGCTTCTTTTCCACATTTTTA** |  |
|  |  | **Sample 5** | **TGTAAAAACTTTATCGATACAAAAATCATTGCAAAAGGCAG-------AAGCTTCTTTTCCACATTTTTA** |  |
|  |  | **Sample 6** | **TGTAAAAACTTTATCGATACAAAAATCATTGCAAAAGGCAG-------AAGCTTCTTTTCCACATTTTTA** |  |

**a) Chromosome that the selected target maps to**

**b) Frequency of the variant allele observed**
